# Isolation and Tissue Distribution of an Insulin-Like Androgenic Gland Hormone (IAG) of the Male Red Deep-Sea Crab, *Chaceon quinquedens*

**DOI:** 10.3390/md15080241

**Published:** 2017-08-01

**Authors:** Amanda Lawrence, Shadaesha Green, Jum Sook Chung

**Affiliations:** Institute of Marine and Environmental Technology, University of Maryland Center for Environmental Science, 701 E. Pratt Street Columbus Center, Baltimore, MD 21202, USA; alawrence@umces.edu (A.L.); sgreen@umces.edu (S.G.)

**Keywords:** *Chaceon quinquedens*, cold water species, red deep-sea crab, insulin-like androgenic gland hormone (IAG), androgenic gland

## Abstract

The insulin-like androgenic gland hormone (IAG) found in decapod crustaceans is known to regulate sexual development in males. IAG is produced in the male-specific endocrine tissue, the androgenic gland (AG); however, IAG expression has been also observed in other tissues of decapod crustacean species including *Callinectes sapidus* and *Scylla paramamosain*. This study aimed to isolate the full-length cDNA sequence of IAG from the AG of male red deep-sea crabs, *Chaceon quinquedens* (*ChqIAG*), and to examine its tissue distribution. To this end, we employed polymerase chain reaction cloning with degenerate primers and 5′ and 3′ rapid amplification of cDNA ends (RACE). The full-length *ChqIAG* cDNA sequence (1555 nt) includes a 366 nt 5′ untranslated region a 453 nt open reading frame encoding 151 amino acids, and a relatively long 3′ UTR of 733 nt. The ORF consists of a 19 aa signal peptide, 32 aa B chain, 56 aa C chain, and 44 aa A chain. The putative ChqIAG amino acid sequence is most similar to those found in other crab species, including *C. sapidus* and *S. paramamosain*, which are clustered together phylogenetically.

## 1. Introduction

Insulin-like androgenic gland hormone (IAG) is produced by the male endocrine organ referred to as the androgenic gland (AG) and is unique to male crustaceans. This gland, first described in the blue crab, *Callinectes sapidus*, is located near the sub-terminal region of the vas deferens, surrounded by muscles of the coxopodite of the last thoracic leg [1]. Following its discovery in *C. sapidus*, AG has been found to be present in other crustacean species including an amphipod species, *Orchestia gammarella* [2].

Androgenic gland hormone (AGH) produced by the AG is known to be involved in male sexual differentiation. The presence of AGH has been supported initially by experiments involving AG manipulations in crustaceans [3]. AG implantation into a female isopod, *Armadillidium vulgae*, resulted in the development of male sexual traits [4]. In female *Cherax quadricarinatus*, AG implantation caused the development of male secondary sexual characteristics including red patching on the propodus and the inhibition of vitellogenesis [5]. With clear evidence of the presence of AGH in crustaceans, its structural elucidation was reported first in the isopod *A. vulgare* [6,7]. The structure of IAGs (=AGHs) share six conserved cysteine residues with that of vertebrate insulin, whereas the rest of the primary sequences show much less similarity [6,7,8,9,10,11].

IAG, being involved in male sexual development, is thought to be exclusively found in male crustaceans. However, it has been reported that IAG has been expressed in tissues aside from the AG. Females of two decapod crab species (*C. sapidus* and *S. paramamosain*) express IAG in both the hepatopancreas and ovary, and male *C. sapidus* show a similar IAG expression in the hepatopancreas [9,10]. In shrimp, IAG expression is reported in the hepatopancreas and nerve cord of *Fenneropenaeus chinensis* in both sexes and the hepatopancreas of *M. nipponense* males [12,13]. Interestingly, in *C. sapidus*, IAG cDNA found in different tissues encode the same putative IAG sequence, while 5′ and 3′ UTR sequences differ [10,14,15]. These findings imply that IAG may be a multifunctional hormone, the function is reported as tissue-specific [14].

The red deep-sea crab, *Chaceon quinquedens*, is an important commercial species in the northeastern United States, where its fishery is federally managed. The *C. quinquedens* fishery is based on the harvesting of males with carapace width exceeding 114 mm as these animals are considered to be adults. *C. quinquedens* are distributed along the continental shelf and slope from Nova Scotia and into the Gulf of Mexico, inhabiting depths from 200 to 1800 m. Compared to warm water crustacean species, much remains unknown about their biology and physiology, including size-related sexual maturity. Like most decapod crustacean species, *C. quinquedens* are sexually dimorphic, females upon maturity exhibit specific morphological features specifically at pubertal molt, such as gonopore formation, ovigerous hair development, etc. [16]. Adult males on the other hand, show no clear external morphological feature(s) distinguishing them from juveniles. With the lack of available knowledge on when and at what size male *C. quinquedens* become sexually mature [17], it is important to garner information on size-related sexual maturity to provide more appropriate regulatory guidelines for male-oriented fisheries.

In this study, we aimed to develop a molecular tool, which can be used for determining the sexual maturity of male *C. quinquedens*. To this end, we isolated the full-length cDNA sequence of male *ChqIAG* from the AG using polymerase chain reaction (PCR) with the degenerate primers, and 5′ and 3′ rapid amplification of cDNA ends (RACE) and examined its tissue distribution in adult male and female *C. quinquedens.*

## 2. Results

### 2.1. cDNA Sequence Analysis

**Figure 1:**
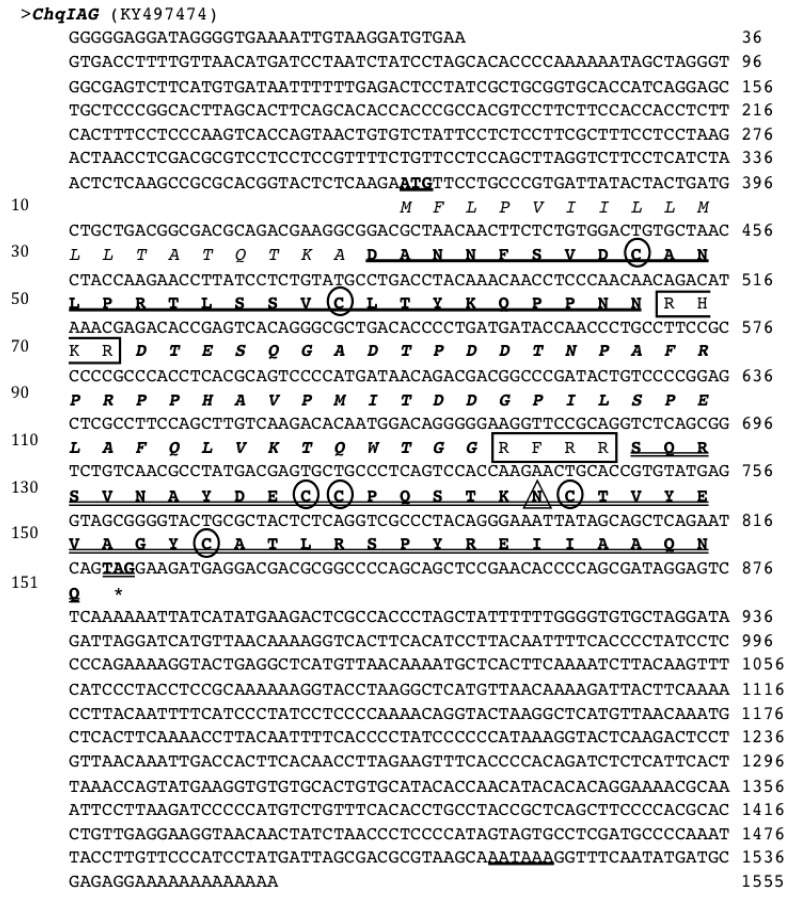
The full-length cDNA and deduced amino acid sequence of ChqIAG isolated from the androgenic gland (AG) of male *C. quinquedens* (GenBank Accession No. KY497474). Predicted signal peptide is italicized. The predicted start codon (ATG) is bolded and underlined, and the stop codon (TAG) is bolded, double underlined and marked with “*”. The nucleotide number is on the right and the amino acid number is on the left hand side of the figure. Two predicted cleavage sites (RHKR/RFRR) are boxed and a polyadenylation signal (AATAAA) is underlined in the 3′ untranslated region (UTR). The six conserved cysteine residues are circled. The B chain is bold and underlined, the C chain is bold and italicized and the A chain is bold and double underlined. The predicted *N*-glycosylation site is noted with a triangle.

The full-length cDNA of *ChqIAG* (GenBank accession number KY497474) was isolated from the AG of an adult male, *C. quinquedens* (Figure 1). The *ChqIAG* cDNA sequence (1555 nt) contains a 366 nt 5′ UTR, a 453 nt ORF encoding 151 amino acids, and a relatively long 3′ UTR of 733 nt. The signal peptide (19 aa): MFLPVIILLMLLTATQTKA was identified (*p* = 0.879) using SignalP (http://www.cbs.dtu.dk/services/SignalP/).

Similar to vertebrate insulin, the putative ChqIAG sequence predicts B and A chains, with six conserved cysteine residues likely involved in three disulfide bridges (two interchains: C_B9_ and C_A12_ and C_B20_ and C_A28_, and one intrachain: C_A11_ and C_A19_). The ChqIAG sequence seen in Figure 1, contains a 19 aa signal peptide, followed by a B chain of 33 aa (underlined), a C chain of 55 aa (in bold and italicized), and an A chain of 44 aa (double underlined). Two cleavage sites, RHKR and RFRR, are located at the end of the B chain and C chain, respectively (boxed). The presence of a putative *N*-glycosylation site at N_A18_ (marked with a triangle) was found in the A chain.

### 2.2. Phylogenetic Tree Analysis

**Figure 2:**
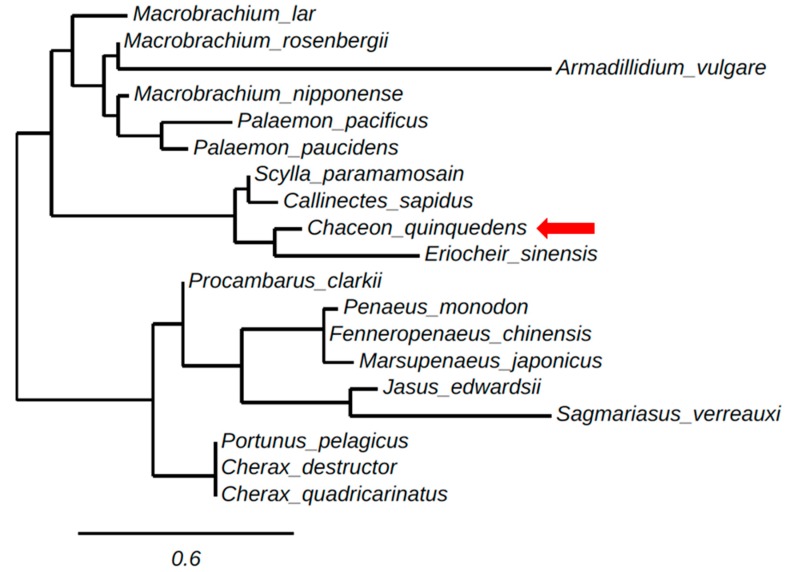
Maximum likelihood tree (using phylogeny.fr) of the open reading frame (ORF) sequence of crustacean insulin-like androgenic gland hormones (IAGs) and androgenic gland hormones (AGHs) obtained from the following species: *C. quinquedens*, KY497474; *M. rosenbergii*, ACJ38227.1; *P. pelagicus*, HM459854; *E. sinensis*, KU724192; *S. paramamosain*, JQ681748.1; *Jasus edwardsii*, KF908794; *Macrobrachium lar*, AB579012.1; *Callinectes sapidus*, KF792074.1; *M. nipponense*, KC460325; *Sagmariasus verreauxi*, KF220491; *Cherax destructor*, ACD91988; *Palaemon pacificus*, AB588014.1; *Procambarus clarkii*, KT343750. *Armadillidium vulgare*, BAA86893.1. *Cherax quadricarinatus*, DQ851163.1; *F. chinensis*, JQ388277.1; *P. monodon*, GU208677.1; *P. paucidens*, AB588013.1; *M. japonicus*, AB598415.1. The scale bar indicates the number of amino acid sequence substitutions per site. Distance represents neighbor-adjoining bootstrap values.

A phylogenetic tree of the ORFs of decapod IAGs and AGH found in isopods was generated using neighbor-adjoining methods, as shown (Figure 2). The phylogenetic tree clusters crustacean IAGs in relative correspondence to their taxonomy. Figure 2 contains two main branches clustering one group including the IAGs of the crayfish, lobsters, and the majority of prawn species, *Jasus edwardsii*, *Sagmariasus verreaux*, *C. destructor*, *P. clarkii, C. quadricarinatus, P. monodon, F. chinensis* and a crab species *Portunus pelagicus*. The other main branch groups crab species; *S. paramamosain, C. quinquedens, C. sapidus* and *Eriocheir sinensis* with a prawn species *M. rosenbergii* and all but one shrimp species *M. japonica*s. *Armadillidium vulgare* as an isopod outgroup clusters away from all malacostraca crustacean species.

### 2.3. Tissue Distribution of ChqIAG

**Figure 3:**
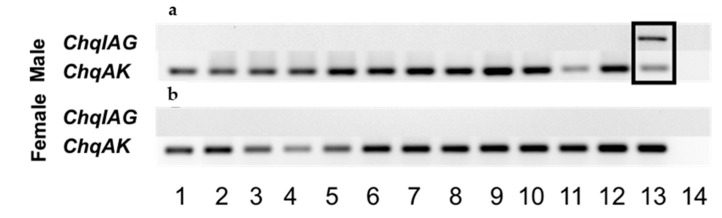
Spatial expression analysis of *ChqIAG* in adult male (**a**) and female (**b**) tissues 1: eyestalk ganglia; 2: thoracic ganglia complex; 3: brain; 4: hypodermis; 5: muscle; 6: gill; 7: midgut; 8: hindgut; 9: heart; 10: hemocytes; 11: hepatopancreas; 12: testis/ovary; 13: androgenic gland/spermatheca; 14: no template control. Arginine kinase (*ChqAK*) was used as a reference gene for amplification.

The spatial distribution of *ChqIAG* expression was examined in various tissues that were dissected from an adult male and female *C. quinquedens* using an end-point reverse-transcriptase polymerase chain reaction (RT-PCR) assay (Promega, Madison, WI, USA). The primers *ChqIAG*-start and -end were used to amplify the entire ORF region of *ChqIAG* (Table 1) (Figure 3a,b, respectively). The AG is the only tissue that expresses *ChqIAG* (Figure 3a, lane 13 boxed). The expression of arginine kinase (AK) as a reference gene is common in all the same tissue cDNA examined. There was no amplification noted by the no template control (lane 14). In contrast to earlier findings in other species, *ChqIAG* expression is absent in the male and female hepatopancreas (Figure 3a, lane 11) as well as the ovary (Figure 3b, Lane 11 and 12, respectively).

### 2.4. Translational Regulatory Sites in ChqIAG cDNA Sequence

Several decapod IAG sequences including that of *C. quinquedens* have been analyzed for putative translational regulatory sites present in 5′ and 3′ UTR using the RegRNA finder (Table 2). *C. quinquedens* share several of these sites with those of other decapod species IAG sequences. Terminal oligopyrimidine tract (TOP) motifs were found in the 5′ and in the 3′ UTRs of the *ChqIAG* sequence*.* An internal ribosome entry site (IRES) and upstream open reading frame (uORF) were present in both the 5′ and 3′ UTRs. A K-Box and Brd-Box were present only in the 5′ UTR of *ChqIAG*. A gamma interferon inhibitor of translation (GAIT element) located in the 3′ UTR of the ceruloplasmin mRNA is only found in the 3′ UTR.

## 3. Discussion

In this study, we isolated the full-length *ChqIAG* cDNA sequence from the androgenic gland of a male red deep-sea crab, *C. quinquedens*, and determined the spatial distribution of its expression in male and female tissues. To examine translational control of IAG, we also analyzed putative translational motifs of IAG sequences in 15 other crustacean species listed from GenBank together with *ChqIAG*.

*ChqIAG* differentiates itself from other crustacean IAG sequences by exhibiting a 733 nt long 3′ UTR, implicating that this region may contain putative translation regulatory sites. The crab species as a group, including *C. sapidus*, *S. paramamosain*, *E. sinensis*, and *C. quinquedens*, have longer 3′ UTRs (~500–830 nt) followed by *P. pelagicus* with a 377 nt 3′ UTR and the lobster, prawn and crayfish species having the smallest 3′ UTR < 300 nt [7]. It is reported that the IAG sequence is possibly encoded by four or more exons separated by three introns: the first one located within the signal peptide, and both the second and third located inside the C peptide [9,18]. The length of the 3′ UTR that is encoded in exon four differs by species. The 3′ UTR length is known to reflect translational efficiency [19], suggesting that *C. quinquedens*’ lengthy 3′ UTR may involve a more proficient translation process.

*ChqIAG* is clustered with three other crab species *S. paramamosain*, *C. sapidus*, and *E. sinensis*. This grouping reflects sequence similarities of the ORFs. The six conserved cysteines within the A and B chains allow for the formation of the intra- and inter-disulfide bonds. The putative amino acid sequence of ChqIAG is most similar to that of *S. paramamosain* and *C. sapidus*, showing 63.6% and 62.9% sequence identity, respectively. *E. sinensis* shares the least sequence identity within the crab cluster at 40.9%. Oddly, the *C. destructor* sequence shows greater similarity to another crab species, *P. pelagicus* than a crayfish belonging to the same genus, *C. quadricarinatus*. The least similar clusters are the prawn/shrimp species cluster and the lobster species cluster at less than ~25% sequence similarity. Overall, IAG sequences isolated from all crab species except for *P. pelagicus* are more similar to each other than those of crayfish or prawn.

IAG is expressed exclusively in the AG of male *C. quinquedens*. It is expected that *ChqIAG* would show a similar expression pattern as that of *C. sapidus* and *S. paramamosain*. However, the exclusive expression of *ChqIAG* expression in the AG is similar to other crustacean species [8] but differs from that of *C. sapidus*. *S. paramamosain*, *F. chinensis*, and *M. nipponense* where IAG has been expressed in both male and female tissues [12,14]. To date, it is still not known if these additional tissues of *S. paramamosain*, *F. chinensis*, and *M. nipponense* contain the same IAG as in the AG as in *C. sapidus* [10]. It is proposed that *C. quinquedens* may have different forms of IAG in these tissues. Hence, they might not have been amplified with the pair of primers used in this study.

The analysis of putative translation regulatory sites in the IAG cDNA sequences shows that *ChqIAG* contains five different motifs: five TOPs, one IRES, one uORF, one K-box and one Brd-box. The presence of IRES in the 5′ UTR of the IAG sequence is unique to *C. quinquedens* and *S. paramamosain*. As another unique feature, *ChqIAG* 5′ UTR also lists a Brd-box which is typically found in the 3′ UTR. The Brd-box found in 3′ UTR is known to down-regulate transcription and subsequently leads to a reduction in protein levels [20]. Having two translational regulatory sites in the 5′ UTR of *ChqIAG* suggests that the translation of IAG levels in AG may be regulated tightly.

Although the ChqIAG cDNA sequence is the only IAG sequence obtained from cold-water species to date, we propose that IRES and Brd-box may be related to ChqIAG translation and may be associated with water temperature. IRES presence is usually associated with the mRNA of growth regulatory genes, which are known to respond to and during environmental stressors [21]. The *ChqIAG* sequence displays the IRES motif more frequently than IAG sequences of any other species in the analysis. Both IRES and box motifs are present among other growth hormone mRNA in cold-water species including *C. japonicus* and *C. opilio* crustacean hyperglycemic hormones [22]. It appears that crustacean species inhabiting colder water are more sensitive to temperature changes than those living in warmer water and may depend on tighter translational control of these neuropeptide hormones than those living in warmer water.

Four different translational regulatory sites are predicted within the *ChqIAG* 3′ UTR, including five TOPs, one uORF, one GAIT, and one IRES. These motifs are most commonly found in the 3′ UTRs of all species in Table 2. The presence of at least three out of four of these regulatory sites present in 14 out of 16 crustaceans in the analysis suggests a more conserved 3′ UTR among IAGs.

There are in general difficulties in distinguishing the sexual maturity of male crustaceans. Unlike adult females, adult males do not exhibit externally visible morphological features. Attempts have been made to determine a relationship between the size and onset of sexual maturity in other deep-sea cold water crustaceans such as the Alaskan tanner crab, *Chionoecetes bairdi*, using chela allometry (using chela height/length, and carapace length/width). However, a clear relationship between size and maturity has not been found yet either in *C. bairdi* [23] or in *C. quinquedens* [17]. Hence it is proposed that establishing a relationship between the size and the levels of crustacean male hormone, IAG, can be used as a tool to define the maturity of male crustaceans.

IAG is known to regulate male sexual development, specifically secondary sexual features and spermatogenesis, suggesting that the levels of IAG expression in the AG and of the hemolymph IAG titers may be closely associated with male sexual maturity. It is reported that IAG requires a binding partner, an insulin-like binding protein, or an insulin-like binding protein like peptide [24]. The exact mode of the IAG mechanism by which it regulates such processes still remains to be studied. With the isolation of the *ChqIAG* cDNA sequence, it will be examined if there is a relationship between the levels of IAGs and the size at sexual maturity of *C. quinquedens* males. This information would provide management agencies guidance for male-driven fisheries that are currently regulated only based on the size, not maturity.

## 4. Materials and Methods

### 4.1. Animal Collection and Maintenance

Adult *C. quinquedens* were collected along the continental shelf off the coast of Virginia in June of 2016 (The Atlantic Red Crab Co., New Bedford, MA, USA). Crabs were captured using baited crab pots and stored in a refrigerated cooling tank onboard the fishing vessel. Adult males and females were then transported in coolers from Newport News, VA to the Institute of Marine and Environmental Technology (IMET, Baltimore, MD, USA). The crabs were kept for three days in a dark, climate-controlled room at 4 °C in 30 ppt artificial seawater. Crabs were chilled on ice prior to dissection. AGs were collected and immediately placed on dry ice. These samples were kept at −80 °C until further processing.

### 4.2. Isolation of the Full-Length ChqIAG cDNA Using PCR with Degenerate Primers

The degenerate primers in Table 1 were based on conserved amino acid sequences of crustacean IAGs [10]. The total RNA was isolated from the AGs of an adult male using the QIAzol lysis reagent (Qiagen, Santa Ana, CA, USA), following the manufacturer’s protocol. The total RNA was quantified using a NanoDrop Lite Spectrophotometer (Thermo Scientific, Waltham, MA, USA). The total RNA (~1–1.5 µg) was subjected to 5′ and 3′ RACE cDNA syntheses using the Switching Mechanism at 5′ End of RNA Template (SMART) cDNA synthesis kit (BD Biosciences, San Jose, CA, USA) following the manufacturer’s protocol. A two-step PCR method was employed as reported [10]. Briefly, the first touch-down polymerase chain reaction (TD-PCR) (BD Biosciences, San Jose, CA, USA) reaction used the degenerate primer dF1 with a universal primer (BD Biosciences) with the following conditions: 94 °C for 2.5 min; eight cycles at 94 °C for 30 s; decreasing annealing temperatures 2 °C/cycle from 42 °C to 39 °C for 30 s; 68 °C for 1.5 min; 27 cycles at 94 °C for 30 s; 43 °C for 30 s; 68 °C for 1.5 min; and the final extension at 68 °C for 7 min. The TD-PCR products served as the template for the semi-nested PCR where dF1 and dR2 primers were used with the following PCR conditions: 94 °C for 2.5 min; 40 cycles at 94 °C for 30 s; 40 °C for 30 s; 72 °C for 1.5 min; followed by a final extension of 72 °C for 7 min. Products from the semi-nested PCR reaction were analyzed on a 1.5% agarose gel. A band of the expected size ~280 bp was excised for DNA extraction. Cloning and sequencing procedures were conducted as described in [13]. From the initially acquired sequencing results of *ChqIAG*, gene-specific primers were generated (see Table 1) for further isolation of the full-length cDNA sequence encoding *ChqIAG*.

### 4.3. 5′ and 3′ RACE of ChqIAG

The initial TD-PCRs for 5′ and 3′ RACE were carried out as described above; however, the gene specific primers *ChqIAG*-5R1 and *ChqIAG*-3F1 were utilized to obtain 5′ and 3′ RACE sequences, respectively. Both 5′ and 3′ RACE TD-PCRs were conducted using the following conditions: 94 °C for 2.5 min; annealing temperatures decreasing 2 °C/cycle from 57 °C to 53 °C for 9 cycles; followed by 27 cycles at 94 °C for 30 s; 58 °C for 30 s; 68 °C for 1.5 min; and the final extension at 68 °C for 7 min. One microliter of the initial TD-PCR product served as the template for the semi-nested PCR and was amplified using nested universal primer (BD Biosciences) and *ChqIAG*-5R2 for 5′ RACE and *ChqIAG*-3F2 for 3′ RACE. Bands with the expected size of ~450–550 bp were excised for DNA extraction (Qiagen), followed by subsequent cloning and sequencing as described in [10].

### 4.4. Sequence Analyses

The ORF finder was used for finding the ORF (www.ncbi.nlm.nih.gov/orffinder/). The putative amino acid ChqIAG was examined for the presence of a signal peptide using Signal P (http://www.cbs.dtu.dk/services/SignalP/). The transcriptional regulatory sites present in the *ChqIAG* sequence were analyzed using RegRNA2.0 (http://regrna2.mbc.nctu.edu.tw/ [25]. The *N*-glycosylation site was determined using (http://www.cbs.dtu.dk/services/NetNGlyc/). A phylogenetic tree was constructed using phylogeny.fr.

### 4.5. Tissue Distribution of ChqIAG Expression

Total RNA was extracted as described above from various tissues from an adult males and females including eyestalk ganglia, thoracic ganglia complex, brain, hypodermis, abdominal muscle, gill, midgut, hindgut, heart, hemocytes, hepatopancreas, testis, ovary, androgenic gland, and spermatheca. One-2 µg of the total RNA of each tissue sample was subjected to the first strand cDNA synthesis using the PrimeScript™ Reverse transcriptase reagent kit with a gDNA eraser (TaKaRa, Mountain View, CA, USA). The *ChqIAG* spatial distribution was determined using an end-point PCR assay. Each tissue’s cDNA (12.5 ng of total RNA equivalent) was amplified with *ChqIAG*-start and end primers (Table 1) under the following PCR conditions: 94 °C for 2.5 min, followed by 30 cycles of 94 °C for 30 s, 60 °C for 30 s, and 72 °C for 1 min, and the final extension at 72 °C for 5 min. As a reference gene, arginine kinase (*ChqAK*) was amplified in the same cDNA samples using *ChqAK*-QF and *ChqAK*-QR primers.

## Figures and Tables

**Table 1:** Primer sequences used for the isolation of the full-length cDNA of *ChqIAG* from the AG of *C. quinquedens* (d = degenerate).

Primer Name	Primer Sequences (5′ → 3′)
dF1	GAYTTYGAYTGYGGNSAYYT
dR2	ACTGCGGCSACMTSGSCGACA
ChqIAG-3F1	ACCAAGAACCTTATCCTCTGTATGCCTGACC
ChqIAG-3F2	GCCCCCGCCCACCTCACGCAGTCCCCATGA
ChqIAG-3F3	AGCGGTCTGTCAACGTGCACGACGAGTGCTGC
ChqIAG-5R1	GCAGCACTCGTCGTGCACGTTGACAGACCGC
ChqIAG-5R2	TGTCCATTGTGTCTTGACAAGCTGGAAGGCGA
ChqIAG-5R3	ATCATCAGGGGTGTCAGCGCCCTGTGACTCGG
ChqIAG-st	ATGTTCCTGCCCGTGATTATACTACTGAT
ChqIAG-end	TACTGATTCTGAGCTGCTATAATTTCCCT
ChqAK-QF	CTGGGCCAGGTATACCGCCGCCTTGTCAGC
ChqAK-QR	GGGGAGCTTGATGTGGACGGAGGCACGCAC

**Table 2:** Putative translational regulatory sites * present in the 5′ (top of the table) and 3′ (bottom of the table) UTRs of decapod crustacean *IAG cDNA* sequences.

	**TOP**	**IRES**	**uORF**	**GAIT Element**	**SECIS**	**ADH_DRE**	**KB**	**CPE**	**GY**	**Brd**	**BRE**
**Species:**											
***C. quinquedens***	5	1	1				1			1	
***M. nipponense***	6										
***P. pelagicus***											
***M. rosenbergii***	9				1						
***E. sinensis***	7								1		
***P. pacificus***	3			3							
***C. destructor***											
***S. verreauxi***	9								1		
***S. paramamosain***	6	1									
***M. japonicus***	4										
***M. lar***	6										
***C. quadricarinatus***	4				2						
***C. sapidus***	3										
***F. chinensis***	5										
***P. paucidens***	2			1							
***P. clarkii***	3										
	**TOP**	**IRES**	**uORF**	**GAIT element**	**SECIS**	**ADH_DRE**	**KB**	**CPE**	**GY**	**Brd**	**BRE**
***C. quinquedens***	5	4	3	1							
***M. nipponense***	24	2	4	1					1	3	
***P. pelagicus***	5	1	2	2					1		
***M. rosenbergii***	31		3	1				1	1	3	
***E. sinensis***	7	3	2	2	1			1			
***P. pacificus***	23		3	1	1					8	
***C. destructor***	3	1	1	2					1		
***S. verreauxi***	9	2	4	6	2						
***S. paramamosain***	2	3	2	6	1					1	
***M. japonicus***	2									1	
***M. lar***	24	2	3	4	1	1		1	1	1	
***C. quadricarinatus***	2	1	2					1		1	
***C. sapidus***	3	2	2	1	1				1		
***F. chinensis***	12	4	2								1
***P. paucidens***	8		2	1							
***P. clarkii***	1										
**motif distribution**	**233**	**27**	**36**	**32**	**10**	**1**	**1**	**4**	**8**	**19**	**1**
**motif frequency**	**0.63**	**0.07**	**0.1**	**0.09**	**0.03**	**0.003**	**0.003**	**0.01**	**0.02**	**0.05**	**0.003**

* Regulatory sites were analyzed using regrna.mbc.nctu.edutw/html/prediction.html: Terminal oligopyrimidine tract (TOP), internal ribosome entry site (IRES), upstream open reading frame (uORF), gamma interferon activated inhibitor of ceruloplasmin mRNA translation (GAIT element), selenocysteine insertion sequence (SECIS), alcohol dehydrogenase 3′ UTR down regulation control element (ADH_DRE), K-box (KB)/cytoplasmic polyadenylation response element (CPE), GY-box (GY), and Brd-box (BRD), Bruno 3′ UTR responsive element (BRE). Motif distribution row represents the number of sequences containing that particular motif (*n* = 16). Motif frequency row represents the frequency of that motif as a percentage of all motifs (*n* = 372). *A. vulagre*, *J. edwardsii* and *P. monodon* are excluded from this figure because only the coding region, not the full-length cDNA sequence of IAGs including 5′ and 3′ UTRs are available in National Center for Biotechnology Information (NCBI).
